# High HIV incidence and low uptake of HIV prevention services: The context of risk for young male adults prior to DREAMS in rural KwaZulu-Natal, South Africa

**DOI:** 10.1371/journal.pone.0208689

**Published:** 2018-12-26

**Authors:** Kathy Baisley, Natsayi Chimbindi, Nondumiso Mthiyane, Sian Floyd, Nuala McGrath, Deenan Pillay, Janet Seeley, Thembelihle Zuma, Jaco Dreyer, Dickman Gareta, Theresa Smit, Tinofa Mutevedzi, Justin Fenty, Kobus Herbst, Isolde Birdthistle, Maryam Shahmanesh

**Affiliations:** 1 Department of Infectious Disease Epidemiology, London School of Hygiene and Tropical Medicine, London, United Kingdom; 2 Africa Health Research Institute, KwaZulu-Natal, South Africa; 3 Africa Health Research Institute, School of Nursing & Public Health, University of KwaZulu-Natal, KwaZulu-Natal, South Africa; 4 Academic Unit of Primary Care and Population Sciences and Department of Social Statistics and Demography, University of Southampton, Southampton, United Kingdom; 5 Research Department of Epidemiology & Public Health, University College London, London, United Kingdom; 6 Division of Infection and Immunity, University College London, London, United Kingdom; 7 Department of Global Health and Development, London School of Hygiene and Tropical Medicine, London, United Kingdom; 8 Department of Population Health, London School of Hygiene and Tropical Medicine, London, United Kingdom; 9 Institute for Global Health, University College London, London, United Kingdom; Instituut voor Tropische Geneeskunde, BELGIUM

## Abstract

**Background:**

Young men are less likely than young women to engage with HIV prevention and care, and their HIV-related mortality is higher. We describe HIV incidence and uptake of HIV services in men 20–29 years(y) in rural KwaZulu-Natal, South Africa, before the roll-out of DREAMS.

**Methods:**

We used data from a population-based demographic and HIV surveillance cohort. HIV incidence was estimated from anonymised testing in an annual serosurvey. Service uptake was assessed in 2011 and 2015, through two self-reported outcomes: 1) HIV testing in the past 12 months(m); 2) voluntary medical male circumcision(VMMC). Logistic regression was used to estimate odds ratios(OR) and 95% confidence intervals(CI) for factors associated with each outcome.

**Results:**

HIV incidence in 2011–2015 was 2.6/100 person-years (95%CI = 2.0–3.4) and 4.2 (95%CI = 3.1–5.6) among men 20-24y and 25-29y, respectively, with no significant change from 2006–2010. N = 1311 and N = 1221 young men participated in the 2011 and 2015 surveys, respectively. In both years, <50% reported testing for HIV in the past 12m. In 2011, only 5% reported VMMC, but coverage in 2015 increased to 40% and 20% in men 20-24y and 25-29y, respectively. HIV testing was positively associated with higher education and mobility. Testing uptake was higher in men reporting >1 partner in the past 12m, or condom use at last sex, but lower in those reporting a casual partner (adjusted (a)OR = 0.53, 95%CI = 0.37–0.75). VMMC uptake was associated with survey year and higher education. Men aged 25-29y and those who were employed (aOR = 0.66; 95%CI = 0.49–0.89) were less likely to report VMMC.

**Conclusions:**

HIV incidence in men 20-29y was very high, and pre-exposure prophylaxis (PrEP) should be considered in this population. Uptake of services was low. VMMC coverage increased dramatically from 2011 to 2015, especially among younger men, suggesting a demand for this service. Interventions designed with and for young men are urgently needed.

## Introduction

Although access to anti-retroviral therapy (ART) has expanded considerably in sub-Saharan Africa (SSA), the number of new HIV infections remains unacceptably high. In SSA, there were an estimated 1.3 million new infections among adults aged ≥15 years in 2015 [1]. Men constitute 44% of these, and 36% of the new infections among young people aged 15–24 years [1]. Although HIV incidence is substantially higher in young women than young men in SSA, young men are twice as likely to die of HIV-related causes than their female counterparts [2,3].

There are strong socioeconomic drivers of the HIV epidemic, and gender inequalities and poverty combine to make adolescent girls and young women particularly vulnerable to HIV infection. However, unequal gender norms also adversely affect men, and in particular, young men. Young men are less likely than women to engage with HIV services across all stages of the HIV prevention and care cascade [4–6]. Studies in SSA have consistently shown that HIV testing coverage is lower among young men than among young women [7]. In a recently-completed Treatment as Prevention (TasP) trial in rural KwaZulu-Natal, South Africa, which included intensive 6-monthly home-based and mobile testing with evening and weekend provision, less than half of eligible men tested[8]. Young men are also less likely to link to care, and to start and stay on ART [4,9,10]. Estimated ART coverage in SSA among HIV positive women aged ≥15 years was 62% in 2016, compared with only 44% among men [11]. In addition to resulting in poorer clinical outcomes, low rates of HIV testing and ART initiation among young men increases the risk of onward transmission to their HIV negative partners.

Stigma, gender norms, perceived negative attitudes of health care providers, and structural barriers to access–such as time and cost of visiting a facility, distance, waiting times and opening hours–remain serious challenges to the delivery of HIV services to young people[12–14]. Young men often have other, more immediate, priorities, such as income generation, developing sexual partnerships, family support, and maintaining social networks. Health facilities are seen as ‘for women’, and health services often reinforce those beliefs, assuming that young men do not need or will not access services [15]. This gender gap in access to health services is a persistent issue that has long been recognised, in SSA and elsewhere [16,17]. However, studies have shown that well-designed interventions that are integrated into the community can engage men to change gender-related attitudes and practices around health care[18–20].

Voluntary medical male circumcision (VMMC) has been shown to reduce the risk of female-to-male HIV transmission by up to 60%, and is recognised as a highly cost-effective HIV prevention intervention [21]. VMMC is only one component in combination HIV prevention, but has advantages in being a single event that does not require ongoing adherence, offers men lifelong benefits, and is a valuable entry point for providing a broader range of health services to men including HIV testing. Since 2007, extensive efforts have been made to scale up VMMC in settings with high HIV prevalence [22]. In 2016, UNAIDS launched a new framework for accelerating HIV prevention and improving the health of adolescent boys and men, with VMMC at its core[23]. The framework has set a target of 90% VMMC coverage for males aged 10–29 years in priority settings, including South Africa. Reaching young men aged 20–29 with VMMC is particularly important, as it will provide some of the greatest efficiency and HIV prevention impact[23,24]. However, achieving the target VMMC coverage will require new service delivery models, and innovative approaches to overcome current barriers that discourage young men from accessing health care.

The DREAMS partnership is an ambitious programme that aims to halt the high incidence of HIV infection among adolescent girls and young women in SSA through rapid scale-up of a combination HIV prevention package to target biological, behavioural, social and structural sources of risk[25]. Whilst the main target population is adolescent girls and young women, the programme also aims to reduce the risk of HIV transmission from male partners. This hinges on improved uptake of HIV testing for male partners and programmes to link those who test positive to HIV treatment, whilst providing VMMC, condom promotion and sexual risk reduction interventions for those who are HIV negative.

This study had two objectives: 1) to describe HIV incidence in young men aged 20–29 years in a hyper-endemic rural area of KwaZulu-Natal, South Africa, where the DREAMS partnership is being implemented; and 2) to describe their uptake of HIV prevention services. HIV prevalence among young men in this area, although lower than in women of the same age, is substantially higher than in other areas of South Africa, and has increased over time [26,27]. When examining uptake of services, we focus on VMMC and HIV testing. These are two components of HIV prevention in male partners of adolescent girls and young women that are being targeted in the DREAMS partnership, and on which we have collected annual data since 2010, allowing us to examine trends over time. We use data from population-based surveys before the roll out of DREAMS, which provides a baseline against which change can be measured after the implementation of DREAMS.

## Methods

### Setting

The African Health Research Institution (AHRI) (formerly the Africa Centre for Health and Population Studies) is a Wellcome-Trust funded research institute in South Africa. Since 2000, household-based surveys have been used to collect demographic data from a population of approximately 100,000 individuals living in an area of 438 km^2^ in rural uMkanyakude District, northern KwaZulu-Natal [28]. Households are surveyed 2–3 times per year, to collect information on birth, deaths and migration patterns for all household members, including non-residents. In addition, since 2003, resident household members who are aged ≥15 years are invited to participate in an annual individual-level survey, which includes an interview on general health and sexual behaviour, and collection of a dried blood spot (DBS) for anonymised HIV testing. DBS results are for research purposes and are not routinely returned to participants. Eligibility lists for the individual-level survey are drawn up based on age and residency status in December of the preceding year. The cohort is one of the world’s largest population-based longitudinal HIV surveillance studies, situated at the epicentre of the dual HIV and TB epidemics, with information on a wide range of health-related and social factors measured over 17 years of follow-up.

### Population

The analysis of HIV incidence uses household demographic and annual HIV serosurvey data from 2006 to 2015, as the period in which ART was widely available in the area. Individuals were included in the analysis if they were male, aged 20–29 years, resident in the surveillance area, participated in the HIV serosurvey at least twice during the period 2006–2015, and their first test was HIV negative.

For the analysis of uptake of HIV prevention and care services (‘service uptake’), all men aged 20–29 years who were resident in the surveillance area and participated in the general health survey in either 2011 or 2015 were included. These time points were chosen to include the most recent survey before the introduction of DREAMS in early 2016, and an earlier survey to examine any secular trends in service uptake before the introduction of DREAMS.

### Statistical methods

Data were entered and verified in an SQL database, and were analysed using Stata 14. HIV incidence rates were calculated as the number of seroconversions per person-year of observation. Person-time was defined from the date the individual first tested negative in the serosurvey until the latest of date of last negative test or seroconversion date. Periods of non-residency (if individuals moved away from the surveillance area and later returned) were excluded from the calculation of person-time. Seroconversion dates were multiply-imputed (using 250 imputations) as a fraction of the interval between the last negative test date and the first positive test date, assuming a uniform distribution. Imputed seroconversion dates which did not fall within a period when the individual was resident in the surveillance area (e.g. if the individual had temporarily out-migrated) were censored at the latest exit date (the end of the period when the individual was last resident) before the imputed seroconversion date, i.e. the seroconversion event did not contribute to the numerator, and person-time after the residency period did not contribute to the denominator. HIV incidence rates (with 95% confidence intervals (CI)) were estimated separately for each age group (aged 20–24 years and 25–29 years) and calendar period (2006–2010 and 2011–2015). To assess trends over time, Poisson regression was used to estimate rate ratios (RR) and 95% CIs for the effect of calendar period on incidence. As a sensitivity analysis, periods of non-residency were included in the calculation of incidence (i.e. all seroconversions contributed to the numerator, and person-time during periods of non-residency contributed to the denominator).

Uptake of services was measured through two self-reported outcomes: 1) having had an HIV test in the past 12 months; and 2) having undergone voluntary medical male circumcision (VMMC). HIV testing was assessed based on the responses to the following questions: a) do you know your HIV status; and if yes, b) when did you last test for HIV. Since DBS results from the annual serosurvey are anonymised and not returned to individuals, participation in the serosurvey was not considered as having tested for HIV. VMMC was assessed based on the responses to the following questions: a) are you circumcised; and if yes, b) where was the procedure conducted; c) was the procedure done for cultural reasons, health reasons, or both. Participants who reported having been circumcised in a medical centre or for medical/health reasons were considered to have undergone VMMC; all others were considered not to have undergone VMMC.

Participant characteristics were tabulated for each survey year, stratified by age group. Logistic regression was used to estimate odds ratios (OR) and 95% CIs for factors associated with uptake of each service; separate models were developed for each outcome. Robust standard errors were used to account for repeat participation by some participants between survey years. Analyses were not weighted for survey non-participation or non-response.

Potential determinants of HIV testing, and of VMMC uptake, were examined using a conceptual framework with three levels: sociodemographic factors, general behavioural factors, and sexual behaviour. For each outcome, age and survey year were considered a priori confounders and were included in all models. First, sociodemographic factors whose age- and survey year-adjusted association with the outcome was significant at p<0.10 were included in a multivariable model; those remaining associated at p<0.10 were retained in a core model. Behavioural factors were then added to this core model one by one. Those that were associated with the outcome at p<0.10, after adjusting for sociodemographic factors were included in a multivariable model and retained if they remained significant at p<0.10. Associations with sexual behaviour factors were subsequently determined in a similar way. Since most of the sexual behaviour questions are only asked of individuals who report having had sex in the past 12 months, the analysis of sexual behaviour was restricted to this sub-group.

### Laboratory methods

Laboratory testing was performed according to manufacturer’s instructions and standard operating procedures in the AHRI laboratories, Durban. DBS were tested for HIV antibody using an enzyme linked immunosorbent assay (ELISA). From 2006‒2008, Vironostika HIV Uni-Form II Ag/Ab (Biomerieux) was used, with the GAC ELISA (Biorad) as a confirmatory test. From 2008–2016, the SD HIV 1/2 ELISA (V3) was used.

### Ethics

Ethical approval for the demographic surveillance study and analyses of these data was granted by the Biomedical Research Ethics Committee of the University of KwaZulu-Natal, South Africa. Separate written informed consent was obtained from all participants for the main household survey, the individual general health and sexual behaviour questionnaires, and the HIV serosurvey. For participants aged <18 years, written informed consent was obtained from the parent/guardian, and written informed assent was obtained from the participant.

## Results

### Survey response rates

#### Participation in the annual HIV serosurvey for HIV incidence and prevalence estimates

During 2006–2015, there were 16,050 young men aged 20–29 who were resident and therefore eligible for at least one of the annual HIV serosurveys; 84% were eligible in more than one survey round. The majority (>90%) were successfully contacted; at the time of contact, 30–45% of individuals were no longer eligible, mostly owing to out-migration. In the survey years from 2006–2011, men aged 25–29 years were less likely than those aged 20–24 to still be eligible at the time of contact; however, from 2012–2015, there was no difference between the age groups in the proportion who remained eligible. Among those who were contacted and still eligible for the survey, annual participations rates ranged from 25%–35% among men aged 20–24 years, and 21%–32% among men aged 25–29. Participation rates in 2011 and 2015 (for HIV prevalence estimates) were 33% and 34% among men aged 20–24 years, respectively, and 29% and 26% among men aged 25–29 years.

Overall, 5223 (45% of 11,605 contacted and still eligible at time of contact) young men ever participated in at least one of the HIV serosurveys. Of those, 90% were HIV negative at the first HIV test, and 93% were eligible in a subsequent survey round. 3311 contributed data to the HIV incidence analysis (i.e. tested at least twice and were HIV negative at the time of the first test).

#### Participation in general health survey for analysis of service uptake

4491 and 4615 young men aged 20–29 were on the eligibility lists for the individual-level surveys in 2011 and 2015, respectively, of whom >90% were contacted. At the time of contact, 1541 (38%) and 1602 (35%) individuals, respectively, were no longer eligible, mostly owing to out-migration.

Among those who were contacted and still eligible for the survey, 1311 (52%) young men in 2011, and 1221 (41%) in 2015, consented to participation in the general health survey; 2218 men participated in only one of the survey years, and 157 participated in both years. In both survey years, men aged 20–24 years were more likely to participate than those aged 25–29 (54% vs 47% in 2011; and 45% vs 35% in 2015; p<0.001). In general, participation rates were lower in urban areas than in rural or peri-urban areas.

### Characteristics of study participants

The majority of survey participants were never married (94% in 2011, and 99% in 2015; Table 1). Overall, 37% of those aged 20–24 and 55% of those aged 25–29 had completed secondary education, with a significant increase between the two survey years. Unemployment was high, with only 17% of those aged 20–24 and 39% of those aged 25–29 having either full- or part-time current employment, and no evidence of a difference between the survey years.

**Table 1:** Characteristics of males aged 20–29 years, by age group and year of survey.

	2011 n (%)		2015 n (%)	
	20–24 years	25–29 years	20–24 years	25–29 years
	*[N = 934]*	*[N = 477]*	*[N = 825]*	*[N = 477]*
**Sociodemographic**				
Marital status				
Never married	860 (95.3%)	414 (90.2%)	794 (100.0%)	449 (98.5%)
Married	0 (0.0%)	5 (1.1%)	0 (0.0%)	4 (0.9%)
Engaged	42 (4.7%)	40 (8.7%)	0 (0.0%)	3 (0.7%)
Education				
None/incomplete primary	34 (4.9%)	31 (8.7%)	26 (3.3%)	17 (3.7%)
Completed primary	13 (1.9%)	15 (4.2%)	15 (1.9%)	9 (2.0%)
Some secondary	429 (61.7%)	133 (37.5%)	427 (53.6%)	160 (35.1%)
Completed secondary	218 (31.4%)	170 (47.9%)	323 (40.6%)	259 (56.8%)
Above secondary	1 (0.1%)	6 (1.7%)	5 (0.6%)	11 (2.4%)
Employment				
Unemployed	558 (81.6%)	222 (60.7%)	667 (83.4%)	283 (61.1%)
Part-time employment	28 (4.1%)	34 (9.3%)	21 (2.6%)	25 (5.4%)
Full-time employment	98 (14.3%)	110 (30.1%)	112 (14.0%)	155 (33.5%)
Residence				
Urban	35 (3.7%)	32 (6.7%)	51 (6.2%)	36 (7.5%)
Peri-urban	354 (37.9%)	199 (41.7%)	308 (37.3%)	198 (41.5%)
Rural	545 (58.4%)	246 (51.6%)	466 (56.5%)	243 (50.9%)
Socioeconomic status				
Low	216 (30.9%)	107 (29.2%)	242 (30.1%)	106 (22.8%)
Middle	205 (29.3%)	113 (30.9%)	316 (39.3%)	207 (44.5%)
High	278 (39.8%)	146 (39.9%)	247 (30.7%)	152 (32.7%)
House connected to electricity supply				
No	140 (19.7%)	69 (18.7%)	63 (7.8%)	16 (3.4%)
Yes	569 (80.3%)	300 (81.3%)	744 (92.2%)	449 (96.6%)
HIV status (% of known)				
Negative	470 (92.0%)	203 (78.1%)	555 (92.8%)	246 (72.4%)
Positive	41 (8.0%)	57 (21.9%)	43 (7.2%)	94 (27.6%)
*Unknown (% of all)*	*423 (45*.*3)*	*217 (45*.*5%)*	*227 (27*.*5%)*	*137 (28*.*7%)*
**Behaviour**				
Previous migration episodes				
None	305 (32.7%)	139 (29.1%)	435 (52.7%)	185 (38.8%)
One	445 (47.6%)	203 (42.6%)	282 (34.2%)	158 (33.1%)
Two or more	184 (19.7%)	135 (28.3%)	108 (13.1%)	134 (28.1%)
Medical circumcision				
No	853 (95.4%)	428 (94.7%)	470 (60.5%)	360 (80.4%)
Yes	41 (4.6%)	24 (5.3%)	307 (39.5%)	88 (19.6%)
Tested for HIV in past 12m				
No	484 (53.6%)	235 (51.4%)	443 (55.8%)	229 (50.3%)
Yes	419 (46.4%)	222 (48.6%)	351 (44.2%)	226 (49.7%)
Knows where to get ART				
No	97 (10.9%)	49 (11.0%)	43 (5.5%)	26 (5.7%)
Yes	793 (89.1%)	396 (89.0%)	744 (94.5%)	427 (94.3%)
Attended a clinic in past 12m				
No	641 (71.0%)	288 (62.9%)	578 (72.9%)	303 (66.9%)
Yes	262 (29.0%)	170 (37.1%)	215 (27.1%)	150 (33.1%)
Sexual partnership pattern				
Never had sex	175 (26.0%)	13 (4.4%)	209 (35.6%)	25 (9.1%)
Not sexually active in past 12m	22 (3.3%)	4 (1.4%)	6 (1.0%)	4 (1.4%)
One partner in past 12m	397 (59.1%)	222 (75.8%)	357 (60.8%)	239 (86.6%)
≥2 partners in past 12m	78 (11.6%)	54 (18.4%)	15 (2.6%)	8 (2.9%)
**Among those reporting sex in past 12m**				
Partner in last 12m AGYW aged 15–19				
No	284 (61.2%)	252 (95.1%)	234 (64.8%)	224 (95.7%)
Yes	180 (38.8%)	13 (4.9%)	127 (35.2%)	10 (4.3%)
Partner in last 12m AGYW aged 20–24				
No	172 (37.1%)	102 (38.5%)	141 (39.1%)	122 (52.1%)
Yes	292 (62.9%)	163 (61.5%)	220 (60.9%)	112 (47.9%)
Used condom at last sex				
No	141 (29.6%)	102 (36.8%)	155 (41.7%)	140 (56.7%)
Yes	336 (70.4%)	175 (63.2%)	217 (58.3%)	107 (43.3%)
Sex without condom in past 12m				
No	233 (48.8%)	104 (37.5%)	129 (34.7%)	55 (22.3%)
Yes	244 (51.2%)	173 (62.5%)	243 (65.3%)	192 (77.7%)
Casual partner in past 12m				
No	370 (77.6%)	228 (82.3%)	311 (83.4%)	225 (91.1%)
Yes	107 (22.4%)	49 (17.7%)	62 (16.6%)	22 (8.9%)

The majority of participants had been members of a household in the surveillance area since the start of the surveillance cohort in 2000 (83% in 2011, and 80% in 2015, with no difference between the age groups). In 2011, 69% of participants had out-migrated from the surveillance area at least once previously and returned; that proportion had reduced to 52% in 2015 (Table 1; p<0.001). Overall, men aged 25–29 were more likely to have out-migrated than those aged 20–24 (66% vs 58%).

Overall, 67% of young men aged 20–24 and 92% of those aged 25–29 reported having had sex in the past year, with a small decline between the survey years in both age groups. Reported condom use declined significantly between the two survey years, in both age groups, with only 52% of sexually active participants reporting condom use at last sex in 2015. Furthermore, condom use was similarly low among men who had undergone VMMC and those who had not (52% vs 53%, respectively).

Over a third (37%) of young men aged 20–24 years reported having a female partner aged 15–19 in the past 12 months, and 62% reported a partner aged 20–24; median partner age difference was –2 years (interquartile range, IQR, –3 to 0), with no evidence of a difference between the two survey years. Only 5% of men aged 25–29 years reported having a female partner aged 15–19, and 55% reported a partner aged 20–24; median partner age difference decreased from –3 years (IQR –4 to –2) in 2011 to –2 years (IQR –4 to –0) in 2015 (p<0.001).

### HIV prevalence and incidence

HIV prevalence among young men aged 20–29 years participating in the HIV serosurvey was 12.7% (95%CI = 10.4%‒15.3%) in 2011 and 14.6% (95%CI = 12.4%‒17.0%) in 2015, and significantly higher among the older age group in both survey years (27.6% vs 7.2% in 2015).

HIV incidence during 2011–2015 was 2.6 per 100 person years (95%CI = 2.0–3.4) among young men 20–24 years and 4.2 (95%CI = 3.1–5.6; Table 2) among those aged 25–29 years. Although incidence in each age group was slightly lower than during 2006–2010, there was no evidence of a significant difference in the rate of new infections between the two calendar periods. However, there was some evidence of a decreasing trend in annual incidence among men aged 20–24 years from 2011 to 2015 (RR for linear trend from one year to the next = 0.86, 95%CI = 0.75–0.97, p = 0.02; S1 Table). In the sensitivity analysis including periods of non-residency, the estimates of incidence were very similar and the conclusions regarding change over time were the same (S2 Table).

**Table 2:** HIV incidence estimates in young men aged 20–29 years, by age group and calendar period.

Age group	Calendar period	New HIV infections	Person-years	Incidence rate / 100 person-years	Rate ratio (95% CI) ^1^
20–24 years	2006–2010	103	3430	2.99 (2.38–3.77)	1
	2011–2015	76	2876	2.62 (2.01–3.42)	0.85 (0.59–1.23)
25–29 years	2006–2010	59	1299	4.54 (3.32–6.21)	1
	2011–2015	66	1592	4.16 (3.12–5.56)	0.92 (0.59–1.42)

^1^Rate ratio comparing HIV incidence in the period 2011–2015 to that in 2006–2015, adjusted for current age.

### Uptake of services

In 2011, less than half (47%) of young men aged 20–29 years reported having tested for HIV in the past 12 months, with no difference between the age groups (Table 1). In 2015, there was some evidence that men aged 25–29 were more likely than those in the younger age group to report having tested (50% vs 45%, p = 0.06).

In 2011, only 5% of young men reported having undergone VMMC, with no difference between the two age groups. By 2015, that proportion had increased significantly, with 40% of those aged 20–24 years and 20% of those 25–29 reporting VMMC.

The majority of participants (89% in 2011 and 94% in 2015) had heard about ART and knew of places where people could get ART if needed. Under a third of participants reported having visited a clinic of any type in the past 12 months, with no difference between the survey years; the proportion was higher among the older than the younger age group (35% vs 28%).

### Factors associated with HIV testing

In the unadjusted analysis, older age, having above primary education, higher socioeconomic status (SES), history of having out-migrated from the survey area, knowledge of where to get ART and being sexually active were positively associated with having tested for HIV in the past 12 months (Table 3). After adjusting for age group and survey year, those associations still remained.

**Table 3:** Factors associated with HIV testing in the past 12 months among young men aged 20–29 years.

	n who know HIV status / N (%)	Unadjusted OR (95% CI)	Age and year adjusted OR (95% CI)	Adjusted OR^1^ (95% CI)
**Sociodemographic**				
Age group		P = 0.07	P = 0.07	P = 0.25
20–24 years	770 / 1697 (45.4%)	1	1	1
25–29 years	448 / 912 (49.1%)	1.16 (0.99–1.37)	1.16 (0.99–1.37)	1.11 (0.93–1.33)
Year		P = 0.63	P = 0.60	P = 0.74
2011	641 / 1360 (47.1%)	1	1	1
2015	577 / 1249 (46.2%)	0.96 (0.83–1.12)	0.96 (0.82–1.12)	0.97 (0.82–1.15)
Residence		P = 0.24	P = 0.28	P = 0.65
Urban	72 / 151 (47.7%)	1	1	1
Peri-urban	493 / 1014 (48.6%)	1.04 (0.74–1.45)	1.04 (0.75–1.45)	1.10 (0.77–1.58)
Rural	653 / 1444 (45.2%)	0.91 (0.65–1.25)	0.92 (0.66–1.27)	1.02 (0.72–1.46)
Education		P = 0.02	P = 0.02	P = 0.02
None/only primary	57 / 154 (37.0%)	1	1	1
Some secondary	509 / 1105 (46.1%)	1.45 (1.03–2.04)	1.49 (1.06–2.09)	1.49 (1.06–2.09)
Completed secondary/above	465 / 954 (48.7%)	1.62 (1.15–2.28)	1.63 (1.15–2.30)	1.63 (1.15–2.30)
Employment		P = 0.39	P = 0.55	P = 0.74
Unemployed	769 / 1668 (46.1%)	1	1	1
Employed (part or full time)	269 / 558 (48.2%)	1.09 (0.90–1.32)	1.06 (0.87–1.29)	1.04 (0.84–1.27)
Socioeconomic status		P = 0.02	P = 0.02	P = 0.11
Low	277 / 645 (42.9%)	1	1	1
Middle	370 / 804 (46.0%)	1.13 (0.92–1.39)	1.13 (0.92–1.39)	1.10 (0.89–1.36)
High	401 / 796 (50.4%)	1.35 (1.10–1.66)	1.34 (1.09–1.65)	1.25 (1.01–1.56)
**Behaviour**				
Previous migration episodes		P = 0.007	P = 0.02	P = 0.07
None	442 / 1020 (43.3%)	1	1	1
One	500 / 1052 (47.5%)	1.18 (1.00–1.41)	1.18 (0.99–1.41)	1.21 (0.96–1.53)
Two or more	276 / 537 (51.4%)	1.38 (1.12–1.70)	1.35 (1.09–1.67)	1.38 (1.03–1.84)
Knows where to get ART		P<0.001	P<0.001	P = 0.41
No	74 / 215 (34.4%)	1	1	1
Yes	1125 / 2360 (47.7%)	1.74 (1.29–2.33)	1.75 (1.30–2.35)	1.18 (0.80–1.75)
Sex in past 12m		P<0.001	P<0.001	P<0.001
No	151 / 457 (33.0%)	1	1	1
Yes	692 / 1362 (50.8%)	2.09 (1.67–2.62)	2.05 (1.63–2.59)	1.98 (1.54–2.55)
**Among those reporting sex in past 12m**				
More than one partner in past 12m		P = 0.02	P = 0.06	P = 0.02
No	758 / 1664 (45.6%)	1	1	1
Yes	84 / 151 (55.6%)	1.50 (1.07–2.10)	1.39 (0.98–1.96)	1.70 (1.08–2.68)
Partner in last 12m AGYW aged 15–24		P = 0.40	P = 0.24	P = 0.57
No	115 / 235 (48.9%)	1	1	1
Yes	560 / 1077 (52.0%)	1.13 (0.85–1.50)	1.22 (0.88–1.68)	1.11 (0.78–1.58)
Used condom at last sex		P<0.001	P<0.001	P<0.001
No	237 / 534 (44.4%)	1	1	1
Yes	454 / 827 (54.9%)	1.53 (1.22–1.90)	1.52 (1.21–1.90)	1.62 (1.27–2.08)
Casual partner in past 12m		P = 0.02	P = 0.01	P<0.001
No	588 / 1125 (52.3%)	1	1	1
Yes	104 / 237 (43.9%)	0.71 (0.54–0.95)	0.70 (0.53–0.93)	0.53 (0.37–0.75)

^1^Sociodemographic variables adjusted for age, year, and education. Behavioural variables adjusted for age, year, education, migration episodes and sex in past 12m. Sexual behaviour among those reporting sex in past 12m adjusted for age, year, education, migration episodes, more than one partner in past 12m, condom use at last sex and having a casual partner.

In the final adjusted analysis, education level was the only sociodemographic factor that remained associated (at p<0.10) with HIV testing (adjusted (a)OR = 1.63, 95%CI = 1.15–2.30, comparing those who had completed secondary with those who had no secondary education). Behavioural factors that remained association with HIV testing were being sexually active (aOR = 1.98, 95%CI = 1.54–2.55), and a history of out-migration (aOR = 1.38, 95%CI = 1.03–1.84, comparing those had out-migrated two or more times with those who had never out-migrated).

Among participants who were sexually active, those who reported >1 partner in the past 12 months, or using a condom at last sex, were more likely to have tested for HIV. Those who reported having had a casual partner in the past 12 months were less likely to have tested (aOR = 0.53, 95%CI = 0.37–0.75)

### Factors associated with VMMC uptake

In the unadjusted analysis, younger age, 2015 survey year, having above primary education, higher SES and knowledge of where to get ART were positively associated with VMMC uptake (Table 4). VMMC uptake was lower among participants who were employed, and those who had a history of having out-migrated from the survey area.

**Table 4:** Factors associated with VMMC uptake among young men aged 20–29 years.

	n circumcised / N (%)	Unadjusted OR (95% CI)	Age and year adjusted OR (95% CI)	Adjusted OR^1^ (95% CI)
**Sociodemographic**				
Age group		P<0.001	P<0.001	P<0.001
20–24 years	348 / 1671 (20.8%)	1	1	1
25–29 years	112 / 900 (12.4%)	0.54 (0.43–0.68)	0.46 (0.36–0.60)	0.47 (0.36–0.62)
Year		P<0.001	P<0.001	P<0.001
2011	65 / 1346 (4.8%)	1	1	1
2015	395 / 1225 (32.2%)	9.38 (7.12–12.35)	9.88 (7.45–13.11)	8.04 (5.95–10.88)
Residence		P = 0.72	P = 0.89	P = 0.89
Urban	30 / 148 (20.3%)	1	1	1
Peri-urban	178 / 995 (17.9%)	0.86 (0.56–1.31)	0.95 (0.60–1.51)	0.89 (0.56–1.43)
Rural	252 / 1428 (17.6%)	0.84 (0.56–1.28)	0.91 (0.58–1.43)	0.89 (0.56–1.42)
Education		P = 0.002	P = 0.03	P = 0.02
None/only primary	13 / 151 (8.6%)	1	1	1
Some secondary	224 / 1093 (20.5%)	2.74 (1.52–4.91)	2.32 (1.22–4.42)	2.27 (1.19–4.33)
Completed secondary/above	199 / 940 (21.2%)	2.85 (1.58–5.13)	2.36 (1.24–4.49)	2.52 (1.32–4.82)
Employment		P<0.001	P = 0.009	P = 0.006
Unemployed	364 / 1648 (22.1%)	1	1	1
Employed (part or full time)	78 / 547 (14.3%)	0.59 (0.45–0.77)	0.68 (0.51–0.91)	0.66 (0.49–0.89)
Socioeconomic status		P = 0.03	P = 0.07	P = 0.16
Low	108 / 636 (17.0%)	1	1	1
Middle	179 / 791 (22.6%)	1.43 (1.09–1.87)	1.30 (0.98–1.73)	1.25 (0.93–1.68)
High	156 / 789 (19.8%)	1.20 (0.92–1.58)	1.38 (1.04–1.85)	1.33 (0.98–1.81)
**Behaviour**				
Previous migration episodes		P = 0.02	P = 0.54	P = 0.48
None	205 / 1003 (20.4%)	1	1	1
One	173 / 1038 (16.7%)	0.78 (0.62–0.97)	1.15 (0.90–1.46)	1.15 (0.89–1.48)
Two or more	82 / 530 (15.5%)	0.71 (0.54–0.94)	1.10 (0.81–1.49)	1.16 (0.84–1.59)
Knows where to get ART		P = 0.003	P = 0.15	P = 0.24
No	20 / 202 (9.9%)	1	1	1
Yes	435 / 2316 (18.8%)	2.10 (1.28–3.45)	1.47 (0.87–2.49)	1.42 (0.80–2.52)
Sex in past 12m		P = 0.68	P = 0.003	P = 0.003
No	82 / 448 (18.3%)	1	1	1
Yes	260 / 1355 (19.2%)	1.06 (0.80–1.40)	1.58 (1.16–2.14)	1.64 (1.19–2.26)
**Among those reporting sex in past 12m**				
More than one partner in past 12m		P = 0.002	P = 0.90	P = 0.42
No	328 / 1647 (19.9%)	1	1	1
Yes	14 / 152 (9.2%)	0.41 (0.23–0.72)	0.96 (0.51–1.80)	0.76 (0.39–1.49)
Partner in last 12m AGYW aged 15–24		P = 0.001	P = 0.007	P = 0.01
No	26 / 230 (11.3%)	1	1	1
Yes	225 / 1078 (20.9%)	2.07 (1.34–3.19)	2.12 (1.23–3.66)	2.08 (1.19–3.67)
Used condom at last sex		P = 0.002	P = 0.09	P = 0.10
No	123 / 530 (23.2%)	1	1	1
Yes	136 / 824 (16.5%)	0.65 (0.50–0.86)	0.77 (0.57–1.04)	0.76 (0.55–1.05)
Casual partner in past 12m		P = 0.46	P = 0.99	P = 0.59
No	219 / 1120 (19.6%)	1	1	1
Yes	41 / 235 (17.4%)	0.87 (0.60–1.26)	1.00 (0.67–1.50)	0.88 (0.57–1.38)

^1^Sociodemographic variables adjusted for age, year, education and employment. Behavioural variables adjusted for age, year, education and employment and sex in past 12m. Sexual behaviour among those reporting sex in past 12m adjusted for age, year, education and employment, condom use at last sex and having an AGYW partner aged 15–24 years.

After adjusting for age group and survey year, the association between education and VMMC uptake still remained, and there was some evidence that those with higher SES were more likely to report VMMC uptake. There was strong evidence that participants who were employed were less likely to report VMMC. There was no longer any evidence of an association with migration history or with knowledge of where to get ART. Although there was no evidence of an association in the crude analysis, after adjusting for age and survey year, participants who were sexually active were more likely to report VMMC uptake.

In the final adjusted analysis, uptake was positively associated with survey year (aOR = 8.04, CI = 5.95–10.88 comparing 2015 with 2011), education above primary (aOR = 2.52, 95%CI = 1.32–4.82 comparing those who completed secondary education or higher with those who had no secondary education), and being sexually active (aOR = 1.64, CI = 1.19–2.26). Older males (aOR = 0.47, CI = 0.36–0.62) and those who were employed (aOR = 0.66, 95%CI = 0.49–0.89) were less likely to report VMMC. Among participants who were sexually active, those who reported a female partner aged 15–24 years were significantly more likely to report VMMC uptake (aOR = 2.08, 95%CI = 1.19–3.67). There was weak evidence that those who used a condom at last sex were less likely to report VMMC (aOR = 0.76, 95%CI = 0.55–1.05, p = 0.10).

After adjusting for all factors in the final model, there was some evidence that males who reported VMMC uptake were less likely to be HIV positive (based on serosurvey results in the same year) than those who did not (aOR = 0.62, 95% CI = 0.35–1.10, p = 0.10). The association was stronger in an analysis restricted to those who were sexually active (aOR = 0.51, 95% CI = 0.26–1.01, p = 0.05).

## Discussion

Overall, our findings indicate that rates of new HIV infections among young men aged 20–29 years in this rural area of KwaZulu-Natal are very high, and although somewhat lower in more recent years, do not appear to be declining significantly. Uptake of available services in general is low, with less than half of men having tested for HIV in the past year, and under a third having visited a clinic. There are encouraging signs in the substantial increase in VMMC uptake between the two survey years, with an 8-fold increase in VMMC uptake among the younger age group. However, despite these increases, VMMC coverage is still well below the UNAIDS target of 90% coverage in males aged 10–29 years [23]. The proportion reporting recent HIV testing is also well below the first ‘90’ of the UNAIDS targets (90% of HIV positive individuals know their status, 90% of those diagnosed are on ART, and 90% of those on ART are virally supressed), and does not appear to have changed over the period 2011 to 2015.

Our results suggest that the focus of the DREAMS partnership on young men primarily as a way to reduce the risk of HIV transmission to adolescent girls and young women may not be sufficient. Instead, the high HIV incidence in young men, and their lack of engagement with sexual and reproductive health services, indicates that the sizeable gaps in uptake of key HIV prevention services among young men should be a priority focus in its own right. There is a need for more innovative prevention and testing methods, and pre-exposure prophylaxis (PrEP) should be considered a priority for men as well as for women. This will ultimately serve to decrease the stigma surrounding the use of PrEP, and increase its preventive potential.

Although overall HIV incidence in men aged 20–29 years during the 5 years from 2011–2015 was not significantly different from that during the preceding 5 years, there was some evidence of a decline in annual incidence during the more recent years in the sub-group of men aged 20–24 years. This may be, in part, a reflection of the increased uptake of VMMC in this age group. A decline in HIV incidence in men aged 15–54 years from 2012 to 2015 in this area has been reported by other authors; they hypothesise that the decline may reflect increased ART uptake and viral suppression in women through the successful roll out of Prevention of Mother to Child Testing option B+, thus protecting men from HIV acquisition [29].

We found that there was some evidence that young men who perceived they were at risk to be more likely to test for HIV, although testing was lower among those who reported a causal partner in the past 12 months. HIV testing was higher among those with a history of out-migration, although it not known whether this reflects increased access to testing in other areas, or increased perception of risk. Less than a third of young men reported having visited any type of clinic in the past year, indicating generally poor engagement with health services. These findings suggest that young men are aware of risk, thus the main bottleneck in service uptake may be the model of service delivery. There is a need for more appropriate local services for young men, and given the high prevalence of HIV in the area, interventions to encourage all sexually active men to test regularly. Further studies may be needed to explore this in more depth.

We also found evidence that some risk behaviours (e.g. condomless sex) appear to be increasing over the period of the two surveys, independent of VMMC. Similar trends have been reported in a population-based South African national survey[26] New approaches may be needed to engage communities in condom provision and enhance the perception of condoms among young men. Although condom distribution in South Africa is higher than in other countries in SSA, with an estimated 6% surplus relative to condom need in 2015, barriers to usage still remain, especially among young people [30,31]. Condom programmes need to be integrated into comprehensive sexual and reproductive health education, and must be responsive to the needs of young people [32].

In the adjusted analysis, we found that young men who were employed, aged 25–29 years, or less educated were less likely to have undergone VMMC. However, unlike HIV testing, there was no evidence of a difference in VMMC uptake between those who had previously out-migrated and those who had remained in the area. Tremendous effort has been put into scaling up of VMMC in 14 priority countries in SSA, including South Africa [22]. Although the coverage of VMMC has expanded rapidly in South Africa, the number of annual circumcisions has remained stable over the last few years, and achieving the target 90% coverage in males aged 10–29 will require innovative measures to expand access and overcome current barriers to services[14,33,34]. The DREAMS partnership includes VMMC as a key element. Male-friendly service delivery approaches–including school-based campaigns, mobile services, extending clinic hours and community delivery of some services–may improve access and uptake [14]. However, delivery models that are convenient for one age group may not be successful with others [35]. Engaging young men in the design and implementation of these programmes will be essential.

Both HIV testing and VMMC uptake were associated with education level, with uptake of each service being highest among young men who had completed secondary education. There has long been an emphasis on increasing educational attainment in girls as an integral part of achieving gender equality, increasing young women’s economic empowerment and reducing their risk of HIV [36]. However, secondary education has a strong impact on the attitudes of men too, and our findings suggest that educational attainment may be equally important for HIV risk reduction in young men.

Strengths of our study include a large population-based survey, with detailed interviews conducted annually and an annual HIV serosurvey. This allowed us to examine trends over time in HIV incidence and in uptake of available HIV services. Our method of calculating HIV incidence censors imputed seroconversion dates that do not occur during a residency episode, and excludes periods of non-residency from the calculation of person-time at risk; however, our findings and conclusions were similar when the non-resident intervals were included, indicating that our results are robust to these assumptions.

Limitations of the study include that HIV testing and VMMC, as well as sexual behaviours, were self-reported in a face-to-face interview, thus may be subject to social desirability bias. We cannot discount the possibility that some of the apparent increase in VMMC uptake between survey years, or difference between the age groups, may be a result of reporting bias. Such bias may also differentially effect younger men, as a result of campaigns aimed at boys and young men to encourage VMMC as the ‘right’ thing to do [34]. Participation rates in any one annual survey were relatively low, although are consistent with reported participation rates in young males in this area [37]. Among those contacted, around a third were no longer eligible at the time of the visit, mostly as a result of having out-migrated. As a result, our findings may not be generalisable to the more mobile groups of the population. As of 2017, eligibility lists will be drawn up throughout the year, so should provide a more accurate picture of current residents. Lastly, the surveys are cross-sectional, so it is difficult to discern the direction of causation.

In summary, we found a very high incidence of HIV among young men aged 20–29 years, with little evidence of decline over the past 10 years. Uptake of HIV testing and other health services was low. Although overall VMMC coverage was less than a third, uptake had increased dramatically over the period 2011 to 2015, especially among men aged 20–24, suggesting a demand for this service. Whilst this shows the potential for DREAMS to have an impact on the health of young men, there is good evidence that designing interventions with young men for young men should be considered, to protect this generation of men at very high risk of HIV acquisition and HIV-related morbidity. The focus of the DREAMS partnership on young men primarily as a way to reduce the risk of HIV transmission to adolescent girls and young women may be a missed opportunity to engage men with health services.

## Supporting information

S1 Table HIV incidence estimates in young men aged 20–29 years, by age group and calendar period (including periods of non-residency). (DOCX)

S2 Table HIV incidence estimates in young men aged 20–29 years, by age group and year, 2006–2016. (DOCX)
