# Supplementary material for: High HIV incidence and low uptake of HIV prevention services: The context of risk for young male adults prior to DREAMS in rural KwaZulu-Natal, South Africa
Source: PLoS One. 2018 Dec 26;13(12):e0208689. doi: 10.1371/journal.pone.0208689 (PMC6306176; doi:10.1371/journal.pone.0208689)
Supplement: S1 Table — (DOCX) [file pone.0208689.s001.docx]

**S1 Table. HIV incidence estimates in young men aged 20–29 years, by age group and calendar period (including periods of non-residency)**

| **Age group** | **Calendar period** | **New HIV infections** | **Person-years** | **Incidence rate / 100 person-years** | **Rate ratio  (95% CI) ^1^** |
| --- | --- | --- | --- | --- | --- |
| 20–24 years | 2006–2010 | 120 | 3897 | 3.08 (2.49 -3.82 ) | 1 |
|  | 2011–2015 | 81 | 3121 | 2.58 (2.00 -3.32 ) | 0.82 (0.58 -1.15 ) |
| 25–29 years | 2006–2010 | 68 | 1530 | 4.43 (3.34 -5.87 ) | 1 |
|  | 2011–2015 | 73 | 1793 | 4.04 (3.07 -5.31 ) | 0.91 (0.61 -1.37 ) |

^1^Rate ratio comparing HIV incidence in the period 2011-2015 to that in 2006-2015, adjusted for current age.
