# Supplementary material for: High HIV incidence and low uptake of HIV prevention services: The context of risk for young male adults prior to DREAMS in rural KwaZulu-Natal, South Africa
Source: PLoS One. 2018 Dec 26;13(12):e0208689. doi: 10.1371/journal.pone.0208689 (PMC6306176; doi:10.1371/journal.pone.0208689)
Supplement: S2 Table — (DOCX) [file pone.0208689.s002.docx]

**S2 Table. HIV incidence estimates in young men aged 20–29 years by age group and year, 2006–2016^1^**

| **Age group** | **Year** | **New HIV infections** | **Person-years** | **Incidence rate / 100 person-years** | **Rate ratio (95% CI) (reference 2006)** | **Linear rate ratio (95% CI) ^2^** |
| --- | --- | --- | --- | --- | --- | --- |
| **20–24 y** | 2006 | 20 | 640 | 3.05 (1.70 -5.48 ) | 1 |  |
|  | 2007 | 21 | 674 | 3.02 (1.74 -5.24 ) | 0.99 (0.42 -2.33 ) | 1.00 (0.84 -1.19 ) |
|  | 2008 | 18 | 694 | 2.50 (1.32 -4.74 ) | 0.82 (0.34 -1.97 ) | P=0.97 |
|  | 2009 | 21 | 694 | 3.01 (1.72 -5.27 ) | 0.99 (0.43 -2.26 ) |  |
|  | 2010 | 23 | 727 | 3.17 (1.87 -5.36 ) | 1.04 (0.48 -2.27 ) |  |
|  | 2011 | 23 | 676 | 3.33 (1.93 -5.76 ) | 1.09 (0.50 -2.41 ) |  |
|  | 2012 | 21 | 624 | 3.36 (1.93 -5.85 ) | 1.10 (0.50 -2.44 ) | 0.86 (0.75 -0.97 ) |
|  | 2013 | 15 | 561 | 2.69 (1.40 -5.15 ) | 0.88 (0.37 -2.13 ) | P=0.02 |
|  | 2014 | 10 | 549 | 1.69 (0.69 -4.14 ) | 0.55 (0.19 -1.64 ) |  |
|  | 2015 | 6 | 466 | 1.27 (0.41 -3.95 ) | 0.42 (0.12 -1.46 ) |  |
| **25–29 y** | 2006 | 9 | 209 | 4.33 (1.84 -10.20) | 1 |  |
|  | 2007 | 12 | 231 | 4.85 (2.20 -10.68) | 1.12 (0.33 -3.83 ) | 0.98 (0.78 -1.24 ) |
|  | 2008 | 12 | 269 | 4.41 (1.98 -9.84 ) | 1.02 (0.32 -3.26 ) | P=0.87 |
|  | 2009 | 12 | 276 | 4.13 (1.83 -9.31 ) | 0.95 (0.29 -3.09 ) |  |
|  | 2010 | 14 | 314 | 4.37 (2.11 -9.04 ) | 1.01 (0.32 -3.15 ) |  |
|  | 2011 | 14 | 346 | 4.04 (2.02 -8.05 ) | 0.93 (0.32 -2.75 ) |  |
|  | 2012 | 14 | 352 | 3.89 (1.87 -8.08 ) | 0.90 (0.29 -2.79 ) | 0.97 (0.84 -1.13 ) |
|  | 2013 | 16 | 331 | 4.82 (2.43 -9.57 ) | 1.11 (0.36 -3.42 ) | P=0.73 |
|  | 2014 | 13 | 310 | 4.02 (1.92 -8.40 ) | 0.93 (0.30 -2.88 ) |  |
|  | 2015 | 9 | 252 | 3.34 (1.32 -8.48 ) | 0.77 (0.22 -2.72 ) |  |

^1^Periods of non-residency are excluded from the calculations. ^2^Rate ratio for linear trend in HIV incidence from one year to the next, between 2006-2010 (unshaded area) and 2011-2015 (shaded area), adjusted for current age. If periods of non-residency are included, RR (95% CI) for trend are as follows: Young men aged 20‒24 years, from 2006‒2010: RR=0.98 (0.83 -1.17); from 2011‒2015: RR=0.81 (0.66 -1.00 ). Young men aged 25‒29 years, from 2006‒2010: RR= 0.98 (0.78 -1.24); from 2011‒2015: RR=0.98 (0.79 -1.20).
